# Functional Gels for Dental Applications: Current Advances and Future Perspectives

**DOI:** 10.3390/gels12100872

**Published:** 2026-09-28

**Authors:** Pedro C. Lopes

**Affiliations:** 1 Faculty of Dental Medicine, Universidade Católica Portuguesa, 3504-505 Viseu, Portugal; paflopes@ucp.pt; 2 Center for Interdisciplinary Research in Health, Universidade Católica Portuguesa, 3504-505 Viseu, Portugal

The continuous evolution of biomaterials has profoundly influenced modern dentistry by providing innovative solutions for disease prevention, minimally invasive treatment, tissue regeneration, and implant rehabilitation. Among these materials, functional gels have emerged as one of the most versatile and rapidly expanding classes of biomaterials. Their unique physicochemical properties—including their high water content, tunable mechanical behaviour, excellent biocompatibility, and ability to incorporate and release bioactive compounds in a controlled manner—have enabled their application across virtually every field of dental medicine. More recently, advances in polymer science, nanotechnology, and bioengineering have further expanded the potential of gel-based systems, transforming them from passive carriers into multifunctional therapeutic platforms capable of actively interacting with the oral environment.

This Special Issue of *Gels*, Functional Gels for Dental Applications, was conceived to provide an updated overview of these developments by bringing together original research and review articles addressing the current challenges and emerging opportunities in this rapidly evolving field. The eight articles published in this Special Issue collectively illustrate the diversity of functional gels and their growing relevance for preventive, restorative, regenerative, and implant dentistry. Although the individual studies explore different clinical applications and material formulations, several common themes emerge, reflecting the direction in which contemporary research is progressing.

One of the most evident trends highlighted throughout this Special Issue is the increasing emphasis on bioactivity. Modern dental materials are no longer expected to perform solely as inert physical barriers or drug carriers; instead, they are increasingly designed to actively promote biological responses, including antimicrobial activity, mineral regeneration, tissue healing, and modulation of the oral microenvironment. This paradigm shift is clearly reflected in several of the contributions included in this Special Issue, where gel formulations incorporate bioactive compounds capable of enhancing therapeutic outcomes while minimizing undesirable side effects.

Preventive dentistry is another key focus of this Special Issue. The prevention and early management of dental diseases continue to be central objectives in clinical practice, and functional gels offer unique advantages for localized treatment due to their prolonged retention within the oral cavity and their capacity for sustained release of active agents. Contributions addressing enamel remineralization, management of white spot lesions, and novel tooth-whitening formulations illustrate how functional gels may simultaneously improve clinical efficacy and preserve dental tissues. The incorporation of naturally derived bioactive compounds and alternative remineralizing agents further reflects the ongoing search for safer and more biologically compatible therapeutic strategies.

Another prominent topic concerns the management of oral microbial biofilms. Biofilm formation remains one of the principal causes of treatment failure in both restorative and implant dentistry, contributing to recurrent caries, peri-implant diseases, and chronic inflammation, and functional gels and gel-derived coatings offer attractive approaches to address this challenge through localized antimicrobial delivery and surface modification strategies. The studies included in this Special Issue demonstrate how engineered gel systems may reduce bacterial adhesion while maintaining favourable biological interactions with surrounding tissues. Such approaches are particularly relevant given the increasing concern regarding antimicrobial resistance and the need for alternative strategies that reduce reliance on conventional antibiotics.

Implant dentistry also benefits from recent advances in functional gel technologies. Surface engineering has become a critical area of research aimed at improving osseointegration while simultaneously reducing bacterial colonization during the critical healing period. Innovative gel-based coatings developed using sol–gel technologies represent promising approaches capable of combining mechanical stability with antimicrobial functionality. These multifunctional surfaces illustrate how material science continues to contribute toward improving the long-term success and predictability of implant therapy.

Patient-centred care constitutes another recurring theme emerging from the published contributions. Conditions such as xerostomia significantly affect patients’ quality of life, influencing oral comfort, nutrition, speech, and susceptibility to oral diseases. Functional gels specifically developed for salivary replacement or stimulation illustrate the potential of biomaterials to address not only disease processes but also symptom management and overall patient wellbeing. Such applications reinforce the importance of designing materials that combine biological performance with ease of clinical use, patient acceptance, and long-term safety.

Beyond their individual clinical applications, the articles included in this Special Issue collectively demonstrate the multidisciplinary nature of functional gel research. Progress in this field increasingly depends on close collaboration between materials scientists, chemists, microbiologists, biomedical engineers, clinicians, and industry partners. The convergence of these disciplines has accelerated the development of increasingly sophisticated materials possessing tailored physicochemical characteristics, controlled degradation profiles, targeted therapeutic activity, and improved clinical performance.

Despite these encouraging advances, important challenges remain before many experimental formulations can be translated into routine clinical practice. One of the principal limitations continues to be the relatively limited availability of robust clinical evidence. While numerous in vitro and preclinical studies demonstrate promising biological performance, well-designed randomized clinical trials with long-term follow-up remain comparatively scarce. Furthermore, considerable heterogeneity in experimental methodologies, material characterization, biological evaluation, and outcome reporting complicates comparisons across studies and limits the establishment of standardized clinical recommendations.

Another important knowledge gap is the long-term stability and durability of many functional gel systems under the complex conditions of oral environments. Factors such as salivary composition, mechanical loading, pH fluctuations, enzymatic degradation, and patient-specific biological variability may substantially influence material performance. Future investigations should therefore emphasize standardized testing protocols that better simulate clinical conditions and facilitate comparison between different formulations.

Looking forward, several emerging research directions are likely to shape the next generation of functional gels for dental applications. Smart or stimuli-responsive hydrogels capable of releasing therapeutic agents in response to environmental triggers—including pH changes, enzymatic activity, temperature, or bacterial metabolites—represent particularly exciting developments. Likewise, nanotechnology continues to offer opportunities for improving drug loading efficiency, targeted delivery, and sustained therapeutic activity while minimizing systemic exposure. The integration of functional gels with regenerative approaches, including stem cell therapies, bioactive molecules, extracellular vesicles, and three-dimensional bioprinting technologies, may further expand their role in oral tissue engineering and regenerative dentistry.

Artificial intelligence and computational materials science are also expected to contribute increasingly to the rational design of future gel formulations. Predictive modelling may facilitate the optimization of polymer compositions, mechanical behaviour, degradation kinetics, and biological performance, accelerating the translation of laboratory discoveries into clinically applicable products. At the same time, the increasing emphasis on sustainability and green chemistry may encourage the development of naturally derived polymers and environmentally responsible manufacturing processes.

Collectively, the contributions published in this Special Issue provide an informative snapshot of the use of functional gels in dentistry. They demonstrate that these materials have evolved well beyond conventional pharmaceutical formulations to become multifunctional biomaterials capable of addressing complex clinical challenges through their bioactivity, controlled delivery, antimicrobial performance, and regenerative potential. While significant work is necessary before many technologies achieve widespread clinical implementation, the scientific progress presented in this Special Issue clearly indicates that functional gels will continue to play an increasingly important role in the future of dental medicine.

As Guest Editors, we sincerely thank all authors for their valuable contributions and the reviewers for their careful evaluation and constructive comments, which have ensured the high quality of this Special Issue. We also express our gratitude to the editorial team of *Gels* for their continuous support throughout the editorial process. We hope that this Special Issue will serve not only as a reference for researchers and clinicians working in dental biomaterials but also as a source of inspiration for future interdisciplinary collaborations and innovative research aimed at improving oral healthcare through advanced functional gel technologies.

